# Congenital Complete Atrioventricular Heart Block in a Pregnant Woman with Sjögren Syndrome: Prenatal Care Follow-Up and the Challenge of Intrauterine Treatment

**DOI:** 10.1055/s-0040-1709738

**Published:** 2020-04

**Authors:** Milene Carvalho Carrilho, Nathalie Jeanne Bravo-Valenzuela, Edward Araujo Júnior

**Affiliations:** 1 Department of Obstetrics, Escola Paulista de Medicina, Universidade Federal de São Paulo, São Paulo, SP, Brazil

**Keywords:** complete congenital heart block, maternal autoantibodies, prenatal diagnosis, prevention, intrauterine treatment, bloqueio cardíaco congênito completo, autoanticorpos maternos, diagnóstico pré-natal, prevenção, tratamento intrauterino

## Abstract

The present report describes a case of complete atrioventricular block (CAVB) diagnosed at 25 weeks of gestation in a pregnant woman with Sjögren's syndrome and positive anti-Ro/SSA antibodies. Fluorinated steroids (dexamethasone and betamethasone) and terbuline were used to increase the fetal heart rate, but the fetal heart block was not reversible, and the administration of drugs was discontinued due to maternal collateral effects. Follow-up fetal echocardiograms were performed, and the fetus evolved with pericardial effusion, presence of fibroelastosis in the right ventricle, and ventricular dysfunction. Interruption of pregnancy by cesarean section was indicated at 34 weeks of gestation, and a cardiac pacemaker was implanted in the male newborn immediately after birth. Therapy for fetuses with CAVB is controversial mainly regarding the use or not of corticosteroids; however, monitoring of the atrioventricular interval by fetal echocardiography should be performed in fetuses from pregnant women with positive autoantibodies anti-Ro/SSA and/or anti-La/SSB to prevent the progression to CAVB.

## Resumo

Este relato descreve um caso de bloqueio atrioventricular completo (BAVC) diagnosticado com 25 semanas de gestação em uma mulher com síndrome de Sjögren e anticorpos anti-Ro/SSA positivos. Esteroides fluoretados (dexametasona e betametasona) e terbulina foram utilizados para aumentar a frequência cardíaca fetal, mas o bloqueio cardíaco fetal não foi reversível, e a administração dos medicamentos foi interrompida devido a efeitos colaterais maternos. Ecocardiogramas fetais de acompanhamento foram realizados, e o feto evoluiu com derrame pericárdico, presença de fibroelastose no ventrículo direito, e disfunção ventricular. A interrupção da gravidez por cesariana foi indicada com 34 semanas, e um marca-passo cardíaco foi implantado no recém-nascido do sexo masculino imediatamente após o nascimento. A terapia para fetos com BAVC é controversa, principalmente no que diz respeito ao uso ou não de corticosteroides; no entanto, o monitoramento do intervalo atrioventricular pela ecocardiografia fetal deve ser feito em fetos de mulheres grávidas com autoanticorpos positivos anti-Ro/SSA e/ou anti-La/SSB para impedir a progressão para o BAVC.

## Introduction

The incidence of atrioventricular block (AVB) is of 1 per 15,000–20,000 live births, and 50% to 55% of these cases are caused by the presence of structural congenital heart disease.^1^ In total, 40% of AVBs are mainly related to SSA/Ro- or SSB/La-positive maternal antibodies. These antibodies may cross the placental circulation and lead to immune-mediated inflammation or fibrosis of fetal conduction cardiac tissue.^2^ ^3^

**Fig. 1:**
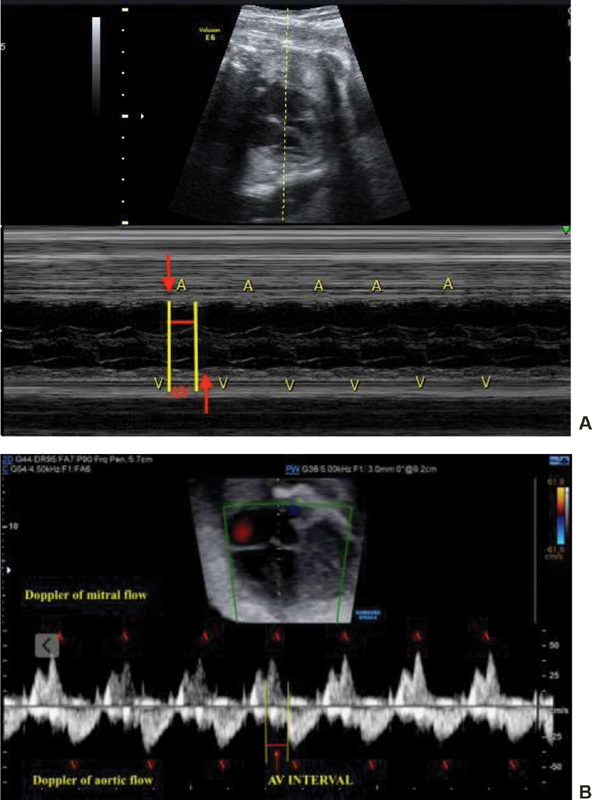
Assessment of the atrioventricular interval time (AV interval, which is analogous to the electrical PR interval) by fetal echocardiogram: M-mode ultrasound (**A**) and Doppler of the mitral and aortic flow (**B**). Abbreviations: A: atrial contraction; V: ventricular contraction.

There are three types of AVB: first, second (incomplete), and third degrees (complete). In first-degree AVB, all electrical impulses are conducted from the atria to the ventricles, with a prolonged atrioventricular (AV) conduction time. In second-degree AVB, not all impulses are conducted from the atria to the ventricles. In complete or third-degree AVB, there is no conduction of electrical impulses from the atria to the ventricles. Thus, complete AVB (CAVB) is characterized by AV dissociation and low ventricular heart rate (HR < 60 bpm) (Fig. 1).^2^ ^3^

In fetuses, the time between atrial and ventricular contractions (AV interval) can be measured using either the ultrasound M-mode (D) or Doppler mode. The AV interval is equivalent to the mechanical PR interval, and it is considered to be extended when its value is above 150 ms. However, the treatment of intrauterine AVB is controversial and involves close monitoring of signs of fetal heart failure.^2^ ^3^ In the present article, we describe a case of AVB associated with maternal autoimmune disease, as well as the guidelines for follow-up and intrauterine treatment.

## Case Report

A 35-year-old primigravida from the city of Sorocaba, Brazil, with Sjögren syndrome and anti-Ro/SSA-positive and anti-La/SSB-negative antibodies was examined at our department with a singleton gestation of 25 weeks; the patient was referred for fetal bradycardia with a heart rate (HR) of 45 bpm for 21 weeks. She had an initial diagnosis of left atrial isomerism associated with interventricular communication. After parental counseling, intrauterine pacemaker placement was indicated; however, the patient refused it. Clinical and echocardiographic follow-ups were conducted, and the mother was treated with corticosteroids and β-adrenergic agents.

**Fig. 2:**
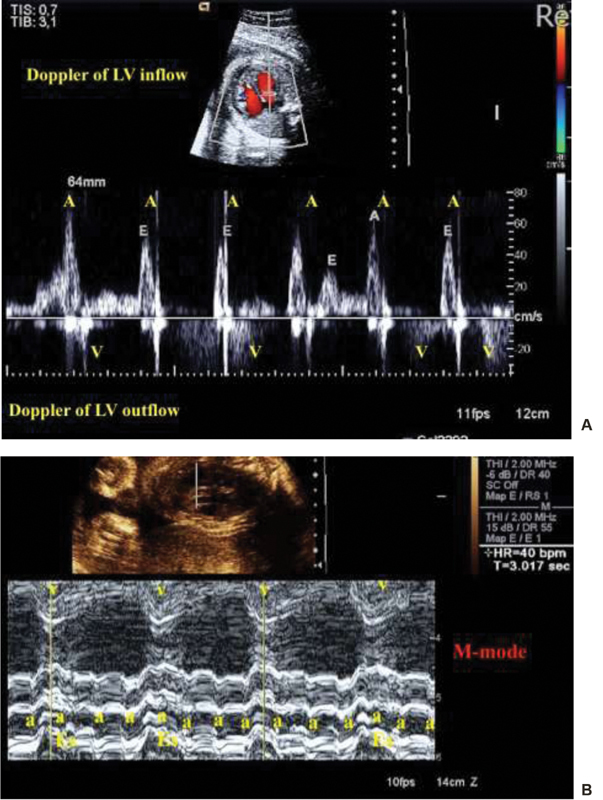
(**A**) Left inflow and outflow tract view showing complete atrioventricular (AV) block by analysis of the inflow and outflow tract Doppler waves. (**B**) M-mode ultrasound showing complete AV block by atrial and ventricular wall movement recordings. Note presence of atrial extrasystoles. Abbreviations: A: atrial contraction; V: ventricular contraction; aEs: atrial extrasystoles; HR: heart rate.

**Fig. 3:**
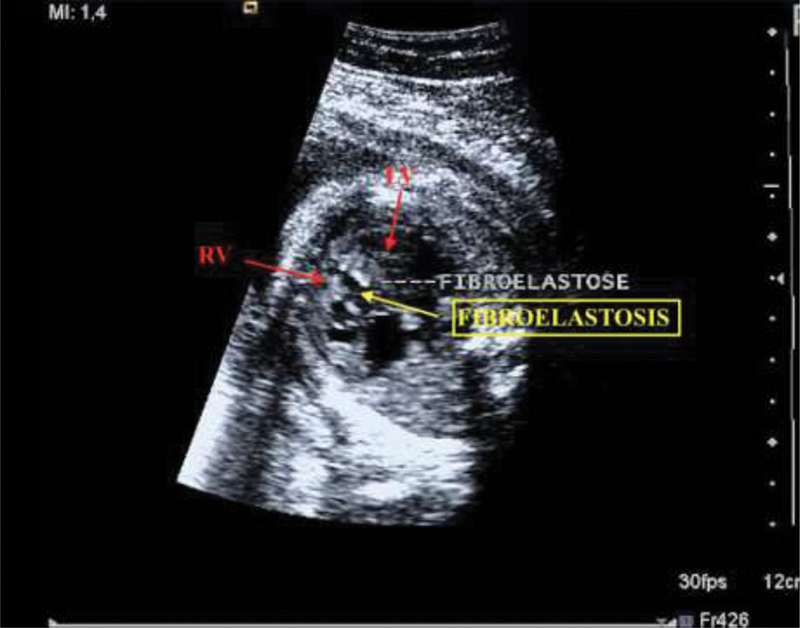
Fetal echocardiogram performed at 32 weeks of gestation showing signs of RV fibroelastosis. Abbreviations: RV: right ventricle, LV: left ventricle; HR: heart rate.

The patient was diagnosed with gestational diabetes mellitus due to corticosteroid use (8 mg/day), and had a HR of 200 bpm associated with symptoms after terbutaline use (400 mg/day). The first fetal echocardiogram, after a protocol to screen for congenital heart disease, showed an anatomically normal heart with bradycardic rhythm, ventricular HR of 40 bpm, and atrial HR of 147 bpm, and a diagnosis of CAVB was made (Fig. 2). The administration of terbutaline was suspended and corticosteroid weaning was started due to the side effects, with improvement in fasting glucose and glycated hemoglobin (HbA1c) levels. At 32 weeks of gestation, the fetus showed effusions on echocardiographic follow-up with discrete pericardial effusion in the lateral wall of the right atrium and suspected right ventricular fibroelastosis (Fig. 3). Another echocardiogram at 34 weeks showed worsening of the fetal pericardial effusion and ventricular dysfunction, and an emergency delivery was indicated. A cesarean section was performed, and a male infant was delivered, weighing 2,240 g, with Apgar scores of 4, 7, and 9 at the 1st, 5^th^, and 10th minutes respectively. The newborn was intubated in the delivery room. Transthoracic echocardiography immediately after birth confirmed normal cardiac anatomy, presence of moderate pericardial effusion without signs of restriction (Fig. 2), and an HR of 40 bpm. Epinephrine was started at a dose of 0.1 µg/kg/min for hemodynamic support, and a 2-hour pacemaker was implanted. The infant's condition was stable, and he was successfully extubated at 5 days of age, after extubation failure due to pulmonary hypertension in the first 24 hours. The infant was discharged in 10 days with an HR of 130 bpm and good clinical condition.

## Discussion

Sjögren syndrome is one of the three most common autoimmune diseases, with a prevalence of 0.1 to 5% of the population,^4^ occurring most often in women, in a 9:1 ratio, with a higher prevalence in the fifth decade of life.^2^ This disease is related to the presence of autoantibodies in 60% of the cases.^3^ These antibodies cause damage to the fetal heart conduction system, leading to CAVB, which, in turn, is associated with a 30% mortality rate.^5^

Complete atrioventricular block is characterized by a complete dissociation between atrial and ventricular activity, with ventricular frequency usually below 60 bpm, in which fetuses with ventricular HR < 55 bpm have a poorer prognosis^6^ due to the presence of endocardial fibroelastosis and signs of fetal hydrops.^7^ ^8^

The follow-up and treatment of these cases is not well-established, and the use of fluorinated corticosteroids (dexamethasone or betamethasone at a dose of 4 to 8 mg/day) is indicated in cases of incomplete AVB (first or second degrees) to prevent progression to CAVB or to achieve incomplete AVB reversal and decrease myocardial fibroelastosis. There is evidence against the use of corticosteroids in cases of CAVB due to the deleterious effects on pregnant women and the presence of irreversible fibrosis in the cardiac conduction tissue of the fetus.^9^ ^10^ ^11^

In the present case, the woman had been using dexamethasone since the diagnosis of CAVB at 23 weeks, without significant change in HR, which was maintained at ∼ 47 bpm. However, the patient developed gestational diabetes mellitus; thus, the administration of the medication was discontinued after a gradual dose reduction. Some studies point to the occurrence of side effects of corticosteroids and recommend that the medication be discontinued in their presence.^2^ ^3^ ^9^ Eliasson et al^9^ performed a multicenter study with 175 patients and demonstrated that, apart from the adverse side effects, the use of corticosteroids showed no beneficial effect in fetuses with HR < 50 bpm or on the presence of hydrops and/or cardiomyopathy.

Some authors recommend the use of intravenous immunoglobulin in cases in which the fetus has systolic cardiac dysfunction and/or signs of endocardial fibroelastosis and/or myocarditis; however, its efficacy has not been proven.^2^ ^3^ Regarding the use of β-sympathomimetics, the patient used terbutaline and developed tachycardia and an estimated HR of 200 bpm, which was associated with symptoms such as malaise and tremors. Yoshida et al^12^ reported the case of a pregnant woman at 22 weeks of gestation with CAVB in which terbutaline was chosen as a β-sympathomimetic drug due to good transplacental passage, to increase the fetal HR and prevent myocardial failure. Another study reported a 10% to 15% increase in fetal HR.^6^ However, despite the increase in fetal HR, there are no studies demonstrating a benefit in terms of survival of these fetuses with this treatment approach.^12^

As there are no studies demonstrating that the use of these medications can modify the survival of these fetuses, pregnancy monitoring was performed every two days after the discontinuation of the corticosteroids and terbutaline, at which time the signs of heart failure were evaluated with the Huhta cardiovascular score^13^ along with HR monitoring.

Percutaneous fetal implantation of a cardiac pacemaker has been shown to be effective in experimental studies, suggesting that it could be used in hydropic human fetuses with CAVB. However, there are no studies that prove its effectiveness in human fetuses.^3^ ^14^

Some authors recommend continuous fetal echocardiographic surveillance from 16 weeks of gestation in pregnant women with Sjögren syndrome or other autoimmune diseases with positive anti-Ro/SSA and/or anti-La/SSB antibodies for the early diagnosis and early treatment of first- and second-degree AVB.^2^ ^3^ ^15^ Patients with anti-Ro/SSA and/or anti-La/SSB antibodies should be advised of the high risk of fetal AVB or neonatal systemic lupus erythematosus, so that the pregnancy can be planned during periods of disease stability, that is, when the patient has low levels of anti-Ro/SSAor anti-La/SSB antibodies. Since 2017, the European League Against Rheumatism (EULAR) has been recommending that hydroxychloroquine should be used during pregnancy, even when the pregnant woman is asymptomatic.^16^

The follow-up and treatment of fetuses with CAVB is still controversial in terms of the use of corticosteroids. However, attention should be paid to the fetuses of pregnant women with positive autoantibodies, with monitoring by fetal echocardiography, to prevent progression to CAVB.
